# How relevant is panallergen sensitization in the development of allergies?

**DOI:** 10.1111/pai.12589

**Published:** 2016-08-22

**Authors:** Olivia E. McKenna, Claudia Asam, Galber R. Araujo, Anargyros Roulias, Luiz R. Goulart, Fatima Ferreira

**Affiliations:** ^1^ Department of Molecular BiologyUniversity of SalzburgSalzburgAustria; ^2^ Laboratory of NanobiotechnologyInstitute of Genetics and BiochemistryFederal University of UberlandiaUberlandiaBrazil; ^3^ Department of Medical Microbiology and ImmunologyUniversity of CaliforniaDavisCAUSA

**Keywords:** allergy diagnosis, allergy treatment, IgE cross‐reactivity, non‐specific lipid transfer proteins, pathogenesis‐related protein family 10, polcalcins, profilins, tropomyosin

## Abstract

Panallergens comprise various protein families of plant as well as animal origin and are responsible for wide IgE cross‐reactivity between related and unrelated allergenic sources. Such cross‐reactivities include reactions between various pollen sources, pollen and plant‐derived foods as well as invertebrate‐derived inhalants and foodstuff. Here, we provide an overview on the most clinically relevant panallergens from plants (profilins, polcalcins, non‐specific lipid transfer proteins, pathogenesis‐related protein family 10 members) and on the prominent animal‐derived panallergen family, tropomyosins. In addition, we explore the role of panallergens in the sensitization process and progress of the allergic disease. Emphasis is given on epidemiological aspects of panallergen sensitization and clinical manifestations. Finally, the issues related to diagnosis and therapy of patients sensitized to panallergens are outlined, and the use of panallergens as predictors for cross‐reactive allergy and as biomarkers for disease severity is discussed.

Allergens are harmless common environmental substances capable of inducing IgE‐mediated (type‐I) hypersensitivities in atopic individuals 1. Allergens have been identified in a variety of sources including over 80 plant species and over 100 animal species 2. In most cases, an allergenic source contains more than one allergen deriving from different protein families, which in turn exhibit differential sensitization rates 3. Many allergens have been implicated in cross‐reactivities, where a patient sensitized to a specific source is also reacting to other related and/or non‐related sources. The foundation of such cross‐recognitions is the presence of conserved linear or conformational IgE epitopes among members of the same protein family 4. Panallergens are proteins that take part in key processes of organisms and are therefore ubiquitously distributed with highly conserved sequences and structures 2. Thus, panallergens are implicated in a multitude of cross‐reactions, even between phylogenetically distant and unrelated organisms 2. Such cross‐reactivities include reactions between various pollen sources 5, 6, pollen and plant foods 7 as well as invertebrate inhalants and foods 8, 9. So far, panallergens have been classified into a few protein families with different levels of distribution and cross‐reactivity. In this review, we provide an overview on the most clinically relevant panallergens from plants (profilins, polcalcins, non‐specific lipid transfer proteins, pathogenesis‐related protein family 10 members) as well as a prominent animal panallergen family (tropomyosins). Until now, 44 profilins, 15 polcalcins, 45 nsLTPs, 24 PR‐10s and 29 tropomyosins have been identified and officially acknowledged by the WHO/IUIS allergen nomenclature subcommittee (www.allergen.org) (Tables 1 and 2).

**Table 1:** List of plant panallergens profilins, polcalcins, nsLTPs and PR‐10s acknowledged by the IUIS nomenclature subcommittee

Species		Protein family			
*Scientific name*	Common name	Profilin	Polcalcin	nsLTP	PR‐10
*Acacia farnesiana*	Needle bush	Aca f 2			
*Actinidia chinensis*	Gold Kiwi fruit			Act c 10	Act c 8
*Actinidia deliciosa*	Kiwi fruit	Act d 9		Act d 10	Act d 8
*Alnus glutinosa*	Alder		Aln g 4		Aln g 1
*Amaranthus retroflexus*	Redroot pigweed	Ama r 2			
*Ambrosia artemisifolia*	Short ragweed	Amb a 8	Amb a 9 Amb a 10	Amb a 6	
*Ananas comosus*	Pineapple	Ana c 1			
*Apium graveolens*	Celery	Api g 4		Api g 2 Api g 6	Api g 1
*Arachis hypogaea*	Peanut	Ara h 5		Ara h 9 Ara h 16 Ara h 17	Ara h 8
*Artemisia vulgaris*	Mugwort	Art v 4	Art v 5	Art v 3	
*Asparagus officinalis*	Asparagus			Aspa o 1	
*Beta vulgaris*	Sugar beet	Beta v 2			
*Betula verrucosa (pendula)*	European white birch	Bet v 2	Bet v 3 Bet v 4		Bet v 1
*Brassica oleracea*	Cabbage			Bra o 3	
*Brassica rapa*	Turnip		Bra r 5		
*Cannabis sativa*	Indian hemp			Can s 3	
*Capsicum annuum*	Bell pepper	Cap a 2			
*Carpinus betulus*	Hornbeam				Car b 1
*Castanea sativa*	Chestnut			Cas s 8	Cas s 1
*Chenopodium album*	Lambsquarters	Che a 2	Che a 3		
*Citrus limon*	Lemon			Cit l 3	
*Citrus retuculata*	Tangerine			Cit r 3	
*Citrus sinensis*	Sweet orange	Cit s 2		Cit s 3	
*Corylus avellana*	Hazel	Cor a 2		Cor a 8	Cor a 1^a^
*Crocus sativus*	Saffron crocus	Cro s 2			
*Cucumis melo*	Muskmelon	Cuc m 2			
*Cynodon dactylon*	Bermuda grass	Cyn d 12	Cyn d 7		
*Daucus carota*	Carrot	Dau c 4			Dau c 1
*Fagus sylvatica*	European beech				Fag s 1
*Fragaria ananassa*	Strawberry	Fra a 4		Fra a 3	Fra a 1
*Glycine max*	Soybean	Gly m 3			Gly m 4
*Helianthus annuus*	Sunflower	Hel a 2		Hel a 3	
*Hevea brasiliensis*	Para rubber tree (latex)	Hev b 8		Hev b 12	
*Hordeum vulgare*	Barley	Hor v 12			
*Juglans regia*	English walnut			Jug r 3	
*Juniperus oxycedrus*	Prickly juniper		Jun o 4		
*Kochia scoparia*	Burning bush	Koc s 2			
*Lactuca sativa*	Cultivated lettuce			Lac s 1	
*Lens culinaris*	Lentil			Len c 3	
*Litchi chinensis*	Litchi	Lit c 1			
*Malus domestica*	Apple	Mal d 4		Mal d 3	Mal d 1
*Mercurialis annua*	Annual mercury	Mer a 1			
*Morus nigra*	Mulberry			Mor n 3	
*Musa acuminata*	Banana	Mus a 1		Mus a 3	
*Olea europea*	Olive	Ole e 2	Ole e 3 Ole e 8	Ole e 7	
*Oryza sativa*	Rice	Ory s 12			
*Ostrya carpinifilia*	European Hophornbeam				Ost c 1
*Parietaria judaica*	Pellitory‐of‐the‐Wall	Par j 3	Par j 4		
*Phaseolus vulgaris*	Green bean			Pha v 3	
*Phleum pratense*	Timothy	Phl p 12	Phl p 7		
*Phoenix dactylifera*	Date palm	Pho d 2			
*Platanus acerifolia*	London plane tree	Pla l 2		Pla a 3	
*Platanus orientalis*	Oriental plane			Pla or 3	
*Prosopis juliflora*	Mesquite	Pro j 2			
*Prunus armeniaca*	Apricot			Pru ar 3	Pru ar 1
*Prunus avium*	Sweet cherry	Pru av 4		Pru av 3	Pru av 1
*Prunus domestica*	European plum			Pru d 3	
*Prunus dulcis*	Almond	Pru du 4		Pru du 3	
*Prunus persica*	Peach	Pru p 4		Pru p 3	Pru p 1
*Punica granatum*	Pomegranate			Pun g 1	
*Pyrus communis*	Pear	Pyr c 4		Pyr c 3	Pyr c 1
*Quercus alba*	White oak				Que a 1
*Rubus idaeus*	Red raspberry			Rub i 3	Rub i 1
*Salsola kali*	Russian thistle	Sal k 4			
*Sinapis alba*	Yellow mustard	Sin a 4		Sin a 3	
*Solanum lycopersicum*	Tomato	Sola l 1		Sola l 3 Sola l 6 Sola l 7	Sola l 4
*Syringa vulgaris*	Lilac		Syr v 3		
*Triticum aestivum*	Wheat	Tri a 12		Tri a 14	
*Vigna radiata*	Mung bean				Vig r 1
*Vitis vinifera*	Grape			Vit v 1	
*Zea mays*	Maize	Zea m 12		Zea m 14	

a Hazel trees contain Cor a 1 as a food allergen in the nut and as an inhalant allergen in the pollen.

**Table 2:** List of tropomyosins acknowledged by the IUIS nomenclature subcommittee

	Species		Protein family
	Scientific name	Common name	Tropomyosin
Arachnids	*Blomia tropicalis*	Mite	Blo t 10
	*Chortoglyphus arcuatus*	Storage mite	Cho a 10
	*Dermatophagoides farinae*	American house dust mite	Der f 10
	*Dermatophagoides pteronyssinus*	European house dust mite	Der p 10
	*Lepidoglyphus destructor*	Storage mite	Lep d 10
	*Tyrophagus putrescentiae*	Storage mite	Tyr p 10
Insects	*Aedes aegypti*	Yellow fever mosquito	Aed a 10
	*Blattella germanica*	German cockroach	Bla g 7
	*Chironomus kiiensis*	Midge	Chi k 10
	*Lepisma saccharina*	Silverfish	Lep s 1
	*Periplaneta americana*	American cockroach	Per a 7
Mollusks	*Helix aspersa*	Brown garden snail	Hel as 1
	*Todarodes pacificus*	Squid	Tod p 1
Parasites	*Anisakis simplex*	Nematode	Ani s 3
	*Ascaris lumbricoides*	Common roundworm	Asc l 3
Seafood	*Charybdis feriatus*	Crab	Cha f 1
	*Crangon crangon*	North Sea shrimp	Cra c 1
	*Homarus americanus*	American lobster	Hom a 1
	*Litopenaeus vannamei*	White shrimp	Lit v 1
	*Macrobrachium rosenbergii*	Giant freshwater prawn	Mac r 1
	*Melicertus latisulcatus*	King prawn	Mel l 1
	*Metapenaeus ensis*	Shrimp	Met e 1
	*Oreochromis mossambicus*	Mozambique tilapia	Ore m 4
	*Pandalus borealis*	Northern shrimp	Pan b 1
	*Panulirus stimpsoni*	Spiny lobster	Pan s 1
	*Penaeus aztecus*	Shrimp	Pen a 1
	*Penaeus indicus*	Shrimp	Pen i 1
	*Penaeus monodon*	Black tiger shrimp	Pen m 1
	*Portunus pelagicus*	Blue swimmer crab	Por p 1

This review also undertakes the task of clarifying the role of panallergens in the sensitization process and the progress of the allergic disease. Whilst controversially discussed, panallergen sensitizations have been shown to provide further complexity to the allergic profile of patients 10. If developed, panallergen sensitization further complicates the allergic disease notably due to its most defining feature, cross‐reactivity. Panallergen cross‐reactivities can result in a wider range of IgE responses to distinct allergen sources, hence creating false positives in diagnosis and further complicating treatment 11. Although just a minority of individuals become sensitized and go onto develop allergy towards panallergens, this phenomenon appears to be highly influenced by many variables including allergen source exposure, geographic differences 12, the type of allergen source and age demography 13. Such variations are reflected in the disparity of results obtained within the literature and highlight the need for understanding panallergens and their sensitization profiles in the development of future diagnostics and treatments of allergy.

## Profilins

Profilins are 12–16 kDa, actin(–monomer)‐binding proteins, expressed in all eukaryotic cells and certain viruses, with the exception of some protists 14, 15. Profilins promote polymerization of actin filaments and monomers and are thus involved in the generation of the cytoskeleton and movement 15. A variety of 50 additional identified profilin ligands, like phosphoinositides or proteins containing proline rich domains (also used for purification), suggest an important role in many more complex molecular processes as well as signal transduction 16, 17. They belong to the α‐β class of proteins with a highly conserved structure consisting of a central antiparallel β‐sheet core surrounded by α‐helices 18. The first allergenic profilin, Bet v 2 from birch pollen, was identified in 1991 19, and since then, many allergenic members have been identified in pollen, plant foods and latex (www.allergen.org). The involvement of profilins in such essential cellular processes explains their ubiquitous expression and high levels of conservation. This, in turn, can justify the strong serologic cross‐reactivity of the molecules 20. The worldwide prevalence of profilin sensitization, which appears to be initiated by sensitization to pollen, lies between 5% and 42%, whilst the sensitization profile is influenced by geographic factors and can vary greatly between different countries 20, 21. For some profilins, the sensitization rates can reach proportions of major allergens >50% (e.g. Pho d 2 from date palm) 22. As profilins are labile against heat and gastric digestion, reactions to food profilins manifested as pollen‐food syndromes are not uncommon 2.

## Polcalcins

Polcalcins are a pollen‐restricted portion of widespread Ca^2+^‐binding proteins comprising a major group of allergenic molecules of this kind. The mostly α‐helical proteins contain the characteristic EF‐hand motifs (helix‐loop‐helix structure), which are responsible for the binding of calcium. Polcalcins have a molecular mass of 9–28 kDa, depending on the number of EF‐hand motifs they hold (2, 3 or 4 EF‐hand motifs), and show a monomeric or dimeric structure. They can assume two different conformations; a closed, Ca^2+^‐free apo‐form and an open, Ca^2+^‐bound holo form which is more stable and results in stronger interactions with IgE antibodies 23. Although the exact physiologic function of polcalcins remains elusive, their localization in the pollen and their ability to regulate intracellular Ca^2+^ levels suggest a contribution in pollen tube outgrowth 24. Allergenic polcalcins derive from tree, grass and weed pollen; are highly cross‐reactive; and induce sensitization rates between 5% and 10% among pollen‐allergic patients 2. It seems that the clinical relevance of polcalcin sensitization is highly dependent on geographic factors and therefore exposure. Phl p 7 from timothy grass pollen is, among all allergenic polcalcins, the most cross‐reactive molecule and could hence be used as a marker to identify multiple pollen sensitizations 25.

## Non‐specific lipid transfer proteins (nsLTPs)

Originally named after their ability to transfer lipid molecules between membranes *in vitro*, which seems unlikely *in vivo*, lipid transfer proteins (LTPs) are widely distributed small and basic proteins in higher plants 26. LTPs can be either lipid specific or can non‐specifically accommodate several classes of lipids (nsLTPs); all allergenic LTP members have been found within the nsLTP cluster. According to their size, nsLTPs can be further subdivided into the 9 kDa nsLTP1 and the 7 kDa nsLTP2 subfamilies 27, 28. Their physiologic function is still unknown; however, accumulating evidence shows that they play a role in cytology, growth and development. Furthermore, the implication of nsLTPs in the general defence of plants classifies them as members of the pathogenesis‐related protein family (PR‐14 family) 26, 29. nsLTPs share a common conserved tertiary structure consisting of four α‐helices stabilized by 4 disulphide bonds (eight‐cysteine motif), forming a hydrophobic tunnel‐like cavity, where interactions with lipids occur 30, 31. Allergenic nsLTPs show a wide distribution in pollen of trees and weeds, in foods (fruits, nuts, seeds and vegetables) as well as in latex 2. Due to their high thermal and proteolytic stability, nsLTPs are potent class I food allergens. Nevertheless, it is still unclear whether pollen or food nsLTPs are the primary sensitizers 32. nsLTPs are clinically highly relevant and cross‐reactive allergens. In Mediterranean areas, nsLTP representatives like Pru p 3 from peach constitute major allergens, whilst in Central and Northern areas, sensitization to Pru p 3 is limited 33.

## Pathogenesis‐related protein family 10 (PR‐10)

PR‐10 proteins function in the general defence mechanisms of plants, and their expression is induced by stress and pathogens. They constitute mostly intracellular, intrinsic and highly similar globular proteins with a length of around 160 amino acids. They share a molecular mass of around 17 kDa and are encoded by a diverse multigene family 34. Their 3D fold consists of 3 α‐helices embedded in an antiparallel β‐sheet consisting of 7 β‐strands forming an amphiphilic intrinsic solvent‐accessible y‐shaped cavity. Crystallographic analyses of Bet v 1 in combination with a variety of different ligands revealed the availability of a promiscuous binding site in the pocket, which could help to unravel the biologic function of PR‐10s 35, 36. Recently, the physiologic ligand quercetin‐3‐O‐sophoroside in birch pollen for Bet v 1 has been identified 37. A subclass of PR‐10 proteins forms a group of pollen and food allergens. The botanically related Fagales species of birch, alder, hornbeam, hop‐hornbeam, hazelnut, beech, chestnut and oak are the main elicitors of early seasonal rhinitis in the temperate climate zone of the Northern Hemisphere. Whilst Bet v 1 is known to act as the main sensitizer, a potential (co)‐sensitization of other Fagales members is hypothesized 38. A high percentage of Fagales pollen‐allergic patients develop oral reactions against a variety of fresh fruits, nuts and vegetables. Such clinical reactions, known as pollen‐food syndrome, are triggered by IgE antibodies, which are produced against aeroallergens and cross‐react with food allergens 7.

## Tropomyosin

**Figure 1:**
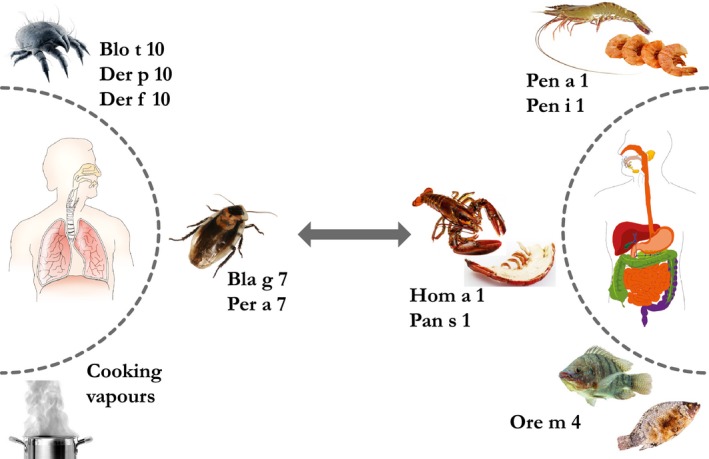
The importance of panallergens in multiple sensitizations. Potential allergic reactions to tropomyosins can occur as a result of primary sensitization via different routes or via IgE cross‐reactivities. The left hand side describes arthropod tropomyosins to which individuals are commonly exposed to via the inhalation route. Cooking vapours of crustaceans are also a possible route of exposure and sensitization. Ingestion of crustaceans and fish, as described in the right hand side, can also cause sensitization to tropomyosins (Table 2 catalogues the mentioned tropomyosins in detail). Images were acquired from ©Ammit; psdesign1/fotolia.com and ©daagron; roblan; suchatbky; tunedin123/123RF.com.

Tropomyosins are α‐helical and, in their native form, dimeric coiled‐coil fibrous structural proteins consisting of two subunits (α, β), each with a molecular weight ranging from 34 to 38 kDa. They form co‐polymers with actin and regulate actin–myosin interactions in skeletal muscles and diversify the functions of actin filaments in different intracellular compartments 39. Evolutionarily, tropomyosins were introduced in metazoa and fungi to expand the functional capacity of actin filaments without enlarging the number of actin isoforms and are therefore not present in plants. In vertebrates, four highly conserved tropomyosin genes are expressed. The increasing number of tropomyosins and splicing variants parallels the increasing complexity of the animal kingdom 15. Tropomyosins are considered important food allergens, whereas tropomyosins from vertebrates are typically non‐allergenic, with the exception of the fresh water fish tilapia 9. Crustacean shellfish form one group of ‘the big‐8’ major food allergens (www.fda.gov). The highly heat‐stable tropomyosins comprise the major allergens in crustaceans and mollusks, making them significant food allergens in exposed populations 40. In addition, these patients show frequent reactions towards (house dust) mites and insects, especially in warmer climates where the growth of house dust mites is favoured. Several studies have already established cross‐reactivity between seafood, mollusks, insects and some parasites 41, 42. However, the route of sensitization remains a matter of debate. There is a controversy as to whether primary sensitization is happening via the gastrointestinal or the respiratory tract. Nonetheless, there are clear indications that inhalation of aeroallergens or cooking vapours is an important sensitising route in the context of tropomyosin hypersensitivity 41, 43. A schematic diagram describing potential exposure routes and sensitization to tropomyosins is presented in Fig. 1.

## Sensitization patterns

Panallergen sensitizations tend to occur as part of a co‐ or poly‐sensitization profile, with panallergen monosensitizations rarely arising 44, 45. In particular, panallergen sensitizations are commonly associated with high or prolonged exposure to a given allergen source, often correlating with increased severity of disease 10, 12. The clinical relevance of panallergens is highly influenced by a number of factors including differences in geographic region 12, allergenic source, the host response against the allergen and exposure 13. Furthermore, differences between child and adult populations have exhibited great variability 12, 46. Hence, studying sensitization profiles of a given source can be a useful tool in understanding the clinical relevance of panallergens. Hatzler et al. exemplifies such a predictable pattern of sensitization for the allergen source *Phleum pratense* (timothy grass). Initial sensitizations towards major (Phl p 1 and Phl p 5) and minor (Phl p 4, 2, 6, 11) allergens gradually result in the onset of allergic disease, following such disease onset the occurrence of panallergen sensitizations, towards Phl p 12 and 7, increases for a minority of patients in correlation with disease severity. Hence, whilst not being an indicative marker for allergic disease onset, panallergen‐specific IgE responses have the potential as clinical biomarkers for increased severity of disease, although it must be emphasized that only a minority of patients become sensitized 10. Such rates of sensitization are influenced by the level of exposure to an allergen source. Feliu et al. demonstrated that children, even with a short disease history, were able to become panallergen sensitized to both date palm profilin (Pho d 2) and peach nsLTP (Pru p 3), with 12% and 13% incidence of IgE positivities, respectively 47. Moreover, high olive pollen rates in southern Spain have been reported to drive increases in sensitizations to the olive nsLTP Ole e 7 12, further showing high exposure rates are strongly correlated with increases in panallergen IgE‐positive patients.

Furthermore, such increased rates of panallergen sensitization have been shown to correlate with an increased severity of allergic symptoms (and in the absence of panallergen allergy). A study carried out by Alverado et al. investigating profilin‐related allergic reactions over both an intense and a mild grass pollen season showed that more severe profilin allergy occurred during the intense season again emphasising the relationship between higher allergen exposures and increased panallergen sensitization rates 44. This phenomenon has been further demonstrated in a large study of 891 allergic patients from Spain where sensitizations to grass pollen profilin correlated with the severity of the allergic disease 12. Conversely, recent studies have emerged describing sensitizations to multiple panallergen families and their effects in reducing the severity of allergic reactions. In particular, such co‐sensitizations have been investigated in peach allergy due to the multiple panallergens present within the fruit, namely Pru p 1 (PR‐10), Pru p 3 (nsLTP) and Pru p 4 (profilin). Patients sensitized to nsLTPs along with PR‐10/profilins present a lower severity of symptoms when compared to patients with sensitizations to nsLTP alone 3. Considering that many allergenic sources contain multiple panallergen families, exploring this avenue and the theories behind such interactions may be of key benefit for developing therapeutic strategies 3, 46. Further investigations into allergies of different plant species have revealed that certain pollen exhibits extraordinarily high rates of panallergen sensitization 48, 49. For *Chenopodium album* pollen (white goosefoot), sensitization rates of 55% and 46% to profilin and polcalcin, respectively, have been reported 48. A further study by Nouri et al., carried out in an Iranian cohort, showed that 81% of patients tested positive to the profilin panallergen Che a 2 49. Disparity of panallergen sensitizations is also exhibited between child and adult populations. Interestingly, Barber et al. also show sensitizations towards the peach nsLTP Pru p 3 to be more prevalent within children than in adult populations in areas of high pollen sensitization in Spain 12. Furthermore, in a Mediterranean study observing nsLTP sensitizations, it was shown that children below the age of six were more frequently sensitized by Pru p 3. However, for the adult population, sensitizations to the walnut nsLTP Jug r 3 reached comparable levels, suggesting an alternative source of sensitization within this age group 46. Such epidemiological differences must be considered when performing clinical investigations, and understanding such profiles of panallergen sensitizations may be of high clinical benefit in both diagnosis and treatment of allergy.

## Panallergen allergy

For only a minority of patients sensitized to panallergens, allergy arises 50. It is in such cases that the cross‐reactivity of panallergens plays a role in worsening the allergic profile of patients via increasing the amount of potential allergenic reactions to allergens in unrelated sources 51. Tropomyosins 43, profilins, PR‐10s and nsLTPs are commonly found in food and plant sources (Tables 1 and 2) and are strongly associated to food allergy arising as a consequence of cross‐reactivity to inhaled allergens 51, 52. Pollen‐food allergy syndrome is occurring commonly for profilins, PR‐10s and nsLTPs 51, especially when exposure rates are increased 12. Associated symptoms range from oral allergy to anaphylaxis and high co‐occurrence with other atopic diseases including allergic rhinitis and asthma, further emphasising the clinical relevance of panallergens 51, 53, 54, 55. Identification of such cross‐reactivities may be vital in finding biomarkers for allergy. For example, the discovery of cross‐reactions between the plane pollen nsLTP Pla a 3 and the peach nsLTP Pru p 3 has elucidated the potential use of Pla a 3 as a biomarker in identifying plane pollen‐allergic patients at risk of developing plant food‐related nsLTP allergies, thereby improving diagnosis and treatment 56. Such strategies may be helpful in avoiding unnecessary dietary restrictions associated to food allergy 57.

It is of note that these different panallergen families present varying levels of severity with regard to the symptoms induced. Tropomyosin represents the only panallergen family with the potential to induce autoimmune responses 58; however, all of the families elicit their effect through the allergic response. Most severely, cases of anaphylaxis have been documented for tropomyosins, nsLTPs 59 and PR‐10s 51, 60. Polcalcins and profilins, whilst presenting slight correlations to anaphylactic reactions, have not been described as direct initiators 61, although wide associations with local reactions (i.e. oral allergy syndrome) have been documented 62. Most frequently, profilins act as minor respiratory allergens resulting in mild allergic symptoms. However, variations in epidemiological factors may present profilin as a potentially severe food allergen and a marker for severe allergy, in particular for grass pollen allergy 44, 47, 63. PR‐10s are associated with both mild and more severe reactions 60, 64, whilst nsLTPs are linked to more severe allergic reactions 65. Polcalcins, whilst exclusively found in pollen sources, have not been implicated in any pollen‐food cross‐reactivities but are more heavily involved in mild respiratory reactions due to inhalation 66.

## Diagnosis and treatment

Panallergen sensitizations may be indicative of allergic disease severity and key in identifying a specific allergenic source, especially when sensitization rates are high 67. Currently, a main treatment of such panallergen allergy is avoidance of the allergy causing foods. This course of action may present detrimental side effects to the patient, with regular intake of fresh fruit having associations with increased health and reduced risk in the development of several chronic diseases 52. For the interests of patients’ quality of life and nutritional satisfaction, it is of benefit to understand the demographics of panallergen sensitization in a specific detail, especially in order to be able to exactly determine the specific allergenic cause. Moreover, with evidence that panallergen sensitizations are linked to increased severity of symptoms, it is crucial to efficiently identify those patients with a higher risk of a severe reaction.

*In vitro* molecular‐based diagnosis (MBD) uses allergenic proteins, either natural, purified or recombinantly produced, to quantify the amount of allergen‐specific IgE antibodies in the blood circulation 59. The use of microarray systems, such as the Immuno‐Sorbent Allergen Chip (ISAC) used for detecting and quantifying the reactivity of specific IgE antibodies towards more than 100 allergens and allergen components, is helpful in the identification and interpretation of allergic sensitization profiles 68. However, it must be noted that IgE positivity alone is not entirely indicative of allergy, and further information obtained by oral/respiratory provocation challenges or basophil activation tests is key in obtaining a true allergic diagnosis 47, 57, 69. Successful diagnosis is essential for deciphering treatment; allergen immunotherapy (AIT) currently represents one of the few ways to modulate allergic disease, and success is highly variable 70. Whilst there is potential in moderating panallergen sensitizations, in particular for food‐related allergy, current commercial availability of therapeutic extracts is limited for minor allergens/panallergens 57. Moreover, lack of standardization provides further difficulty in the use of commercial extracts 11, 71.

Exploration of the hierarchical patterns of cross‐reactive molecules may also provide further insight into panallergen sensitizations and potentially elucidate novel therapeutics based on the concept that some molecules are more cross‐reactive than others. A recent microarray study by Pfiffner et al. investigates the structural basis of cross‐reactivity using an iterative motif detection algorithm 72. Identifying such hierarchies raises the question as to whether treatments using AIT should constitute of more frequently cross‐reacting allergens or an allergen that less frequently cross‐reacts with other allergens, but targets a larger repertoire of allergens within the hierarchy. Further exploration into individual panallergen sensitization profiles (e.g. nsLTPs) 46 may provide key insight into the mechanisms of panallergens in allergy and reveal predicable patterns of cross‐reactivities, thus improving diagnosis. Furthermore, using panallergens as biomarkers for disease severity and as predictors of cross‐reactive allergens may pose the most clinically relevant use of panallergens in allergic disease 47, 69.

## Summary and conclusions

In daily clinical practice, it is often observed that individuals allergic to fruits, nuts and vegetables have sensitivities to more than one food, not necessarily belonging to the same family. In addition, many of them are also allergic to pollens and/or seafood. These associations occur when the same IgE antibody is able to recognize similar epitopes in different panallergens and appear to be highly influenced by many variables. This phenomenon explains why some patients develop a severe allergic reaction upon ingesting allergenic foods they have never before encountered 73. Cross‐reactivity can also occur due to conformational diversity of antibodies 74 and through T‐cell epitopes 75, which in turn may lead to disease. Multiple reactivity to inhalant or allergenic food sources appeared to be caused by the sensitization to at least one representative molecule from a single panallergen group, emphasising the importance of panallergens in multiple sensitizations (Fig. 1).

In conclusion, panallergens comprise a variety of protein families of plant and animal origin and are responsible for wide IgE cross‐reactivity between different allergenic sources. Panallergen sensitizations are clinical ‘warning signs’ for elevated risk of multiple allergies as well as severe symptoms. Thus, improving the understanding of this immunologic cross‐reactivity is essential for the interpretation of their clinical relevance, the development of improved diagnostic tests and the generation of more efficacious immunotherapy agents. Future studies focusing on the identification and evaluation of cross‐reactive epitopes between different members of panallergenic families might provide the basis for understanding their role in sensitization and allergic reactions.
